# Climate and health capacity building for climate-resilient health systems: a case study of the ASPHER-GCCHE European course

**DOI:** 10.1093/eurpub/ckag193

**Published:** 2026-10-27

**Authors:** Eric P Twomey, Danielly de P Magalhães, Chiara Cadeddu, Nicola Hamacher, Margaret O’Donnell, Lore Leighton, Robert Otok, Laurent Chambaud, Cecilia Sorensen, Ana-Catarina Pinho-Gomes

**Affiliations:** ASPHER—The Association of Schools of Public Health in the European Region, Brussels, Belgium; Department of Epidemiology and Public Health, Swiss Tropical and Public Health Institute, Allschwil, Switzerland; Swiss Tropical And Public Health Institute, University of Basel, Basel, Switzerland; GCCHE—Global Consortium on Climate and Health Education, Mailman School of Public Health, Columbia University, New York City, NY, United States; Erasmus School of Health Policy and Management, Erasmus University Rotterdam, Rotterdam, The Netherlands; GCCHE—Global Consortium on Climate and Health Education, Mailman School of Public Health, Columbia University, New York City, NY, United States; GCCHE—Global Consortium on Climate and Health Education, Mailman School of Public Health, Columbia University, New York City, NY, United States; ASPHER—The Association of Schools of Public Health in the European Region, Brussels, Belgium; ASPHER—The Association of Schools of Public Health in the European Region, Brussels, Belgium; ASPHER—The Association of Schools of Public Health in the European Region, Brussels, Belgium; GCCHE—Global Consortium on Climate and Health Education, Mailman School of Public Health, Columbia University, New York City, NY, United States; Institute for Global Health, University College London, London, United Kingdom

## Abstract

Climate change poses growing risks to population health and health system functioning across Europe. Building climate-resilient and low-carbon health systems requires a workforce equipped with appropriate competencies. However, structured climate and health education remains unevenly implemented. This study evaluated the 2025 ASPHER–GCCHE European Climate Resilient Health Systems Course, a large-scale, competency-based online training programme designed to strengthen climate-health capacity among health professionals. We conducted a longitudinal evaluation of a 10-week, open-access, fully virtual course delivered in English between January and April 2025. Participants completed pre- and post-course surveys assessing self-reported confidence across six climate-resilience competency domains using a 10-point Likert scale. Paired Wilcoxon signed-rank tests assessed pre–post changes overall and by geographical region. A total of 4432 individuals from 164 countries registered for the course. Among 1029 participants (1029) from 119 countries, 446 participants were from the WHO European Region, met attendance and assessment criteria and were included in the analysis. Statistically significant improvements were observed across all six competency domains (*P < .*001), with mean confidence increases ranging from 45% to 59%. Self-reported confidence gains were consistent across European subregions, indicating comparable learning effects despite differing policy and institutional contexts. Nearly half of all registrants were from low- and middle-income countries, highlighting the reach of the open-access model. This evaluation demonstrates that competency-based online education was associated with improved self-reported confidence in climate-resilient health-system competencies. Integrating similar training into formal education and professional development pathways may support implementation of climate-resilient and low-carbon health system policies across Europe and beyond.

## Introduction

Climate change is increasingly recognized as a major threat to population health. The World Health Organization (WHO) and the World Economic Forum estimate that climate-related exposures may cause between 250 000 and 725 000 additional deaths annually between 2030 and 2050, driven primarily by heat stress, diarrhoeal diseases, malaria, and undernutrition [1, 2]. Europe is not exempt from these trends: heat-related mortality continues to rise, with an estimated 62 775 premature deaths in 2024 alone [3]. At the same time, many European health systems remain insufficiently prepared to respond to mounting climate-related pressures including heat-related surges in morbidity and mortality, disruptions to facilities and supply chains during floods, wildfires and storms, shifting infectious-disease risks, and increased demand for emergency preparedness and continuity planning [4].

Health systems are on the frontline of climate change. They face growing disease burdens and increasing disruption from extreme weather events, while simultaneously contributing to environmental degradation and greenhouse gas emissions [1]. Climate-related risks to health systems vary by geographic context, hazard type, exposure, and underlying vulnerability, including socioeconomic conditions and access to care [5]. Healthcare systems account for approximately 5% of global carbon emissions, with a substantial share originating from high-income regions, such as Europe [6]. Evidence suggests that health systems that implement structural and operational reforms to address climate risks can improve resilience while reducing environmental impact and operational costs [7].

These challenges underscore the need to equip health professionals with competencies to anticipate, prepare, and respond to climate-related health risks. This requires educational approaches that move beyond awareness towards structured, competency-based training capable of shaping professional practice, institutional responses, and policy. In this context, climate change and health (C&H) education, defined as the systematic integration of climate-related knowledge, skills, and competencies into health and public health training, has gained increasing prominence. International and regional policy frameworks, including the European Green Deal, WHO’s Alliance for Transformative Action on Climate and Health (ATACH), and the declarations of the Seventh Ministerial Conference on Environment and Health, explicitly call for strengthened climate-related education for health professionals [8, 9].

Despite this policy momentum, the provision of C&H education remains uneven and fragmented, particularly in Europe. A recent global survey found that while 70% of public health institutions worldwide reported offering some form of C&H education, only 39% made such training mandatory [10]. In Europe, only 41% of schools of public health were found providing C&H content [11].

Strengthening the climate-related competencies of the public health workforce is central to building climate-resilient health systems. Public health professionals play a key role in translating evidence into policy, implementing adaptation strategies, and protecting vulnerable populations [10]. Enhancing workforce capacity supports emergency preparedness, continuity of essential services during climate shocks, and recovery following extreme events, while enabling health systems to contribute to broader mitigation and sustainability goals.

Several initiatives have begun to address gaps in C&H education. The Global Consortium on Climate and Health Education (GCCHE), hosted by Columbia University, has led the development of structured training programmes, including the Pan-American Climate Resilient Health Systems Course, which demonstrated significant improvements in participants’ self-reported competencies [12]. In Europe, the European Climate and Health Responder Course, delivered in 2024 through a collaboration between GCCHE and the Association of Schools of Public Health in the European Region (ASPHER), similarly reported substantial self-reported confidence gains across domains including climate-health communication, emergency preparedness, adaptation, and mitigation-related practice [13]. While promising, many existing initiatives remain limited in scale, language accessibility, and institutional integration, and evaluations frequently focus on participation metrics rather than systematic assessment of learning outcomes.

In early 2025, ASPHER and GCCHE—supported by WHO and the European Environment Agency—launched the European Climate Resilient Health Systems Course as a successor to the 2024 programme. This open-access, fully virtual, and certified course comprised 10 sessions with a 90-minute duration and combined expert-led lectures with applied case studies, focusing on health system resilience, decarbonization, adaptation, and sustainability. The programme was designed to build both foundational and applied competencies through interactive learning, structured assessments, and defined certification criteria.

To our knowledge, this study presents the first structured evaluation of a certified, pan-European, competency-based C&H education initiative focused explicitly on climate-resilient health systems. Beyond documenting participation, it evaluates changes in participants’ self-reported confidence across key competency domains and aims to:

- describe the reach, structure, and content of the 2025 ASPHER–GCCHE open-access course;
- assess pre–post changes in self-reported confidence among matched respondents in Europe and explore differences across EU-27, EEA-32, and WHO European Region;
- identify gaps and lessons for scaling C&H education in Europe; and
- position the initiative within the broader policy and academic landscape as a replicable model for other regions.

## Methods

### Study design and course structure

The curriculum (detailed curriculum provided in Supplementary Text S1) and learning objectives were co-developed by ASPHER and GCCHE, aligned with the GCCHE Core Competencies and ASPHER Core Curriculum for preparing health professionals to respond to the climate crisis and the WHO–ASPHER Competency Framework for the Public Health Workforce in the European Region [10, 14, 15]. Learning objectives were structured using Bloom’s taxonomy to address diverse participant backgrounds and to progress from foundational knowledge to applied and analytical competencies [16].

Each of the ten 90-minute sessions comprised a 40-minute conceptual lecture followed by two 15-minute case-study presentations, designed to support translation of theory into practice. Case studies illustrated climate and health action across European contexts, with a focus on adaptation and sustainability.

The curriculum addressed assessment of health system vulnerabilities and adaptation options, measurement and monitoring of climate resilience and environmental sustainability, and implementation of health system greening and decarbonization, while integrating health equity and stakeholder engagement.

All lectures and case studies were delivered by European experts, selected to ensure geographic representation and gender balance. Contributors represented governmental, intergovernmental, non-governmental, and academic institutions, including the World Health Organization (WHO), European Environment Agency (EEA), Organization for Economic Co-operation and Development (OECD), International Association of National Public Health Institutes (IANPHI), European Centre for Disease Prevention and Control (ECDC), National Institute for Health and Care Excellence (NICE), Lancet Countdown Europe, and multiple European universities.

The course consisted of 10 live online sessions delivered via Zoom, held weekly from the 28th of January to the 8th of April 2025. It was offered in English, free of charge, and open to participants worldwide. Recorded sessions are publicly available [17].

### Audience, participant recruitment, and registration

The course was promoted through the ASPHER and GCCHE networks. The primary target audience included Europe-based public health students and professionals, health system planners and administrators, sustainability officers, emergency planners, clinicians, and allied health professionals. To maximise access, enrolment was free and open to all interested individuals regardless of geographic location. Although the course was designed around European policy frameworks, case studies and institutional partners, registration was open globally. We therefore report global reach descriptively while analysing European policy-relevant subgroups (EU-27, EEA-32, and WHO European Region) to assess whether learning gains differed across overlapping European governance contexts.

At registration, participants completed a demographic form capturing country of residence, professional background and characteristics, employment sector, prior climate and health training (yes/no), and student status (yes/no).

### Longitudinal survey

Participants completed a pre-course survey at registration and a post-course survey alongside the final examination. The study protocol was classified as exempt by Columbia University’s Institutional Review Board (AAAR4912).

The longitudinal survey assessed self-reported confidence across six climate and health competency domains using a 10-point Likert scale (1 = not confident/skilled; 10 = very confident/skilled):

- Communicating about health system decarbonization and climate resilience
- Participating in vulnerability and adaptation assessments
- Contributing to resilience-building and reduction of health system environmental footprints
- Applying vulnerability and adaptation frameworks
- Supporting community and health system adaptation to climate-related threats
- Developing climate resilience plans

### Course completion requirements and certification

Certification was granted to participants who attended at least 7 of 10 live sessions and achieved a minimum score of 70% on a final multiple-choice examination. Attendance was verified using Zoom reports linked to registered email addresses. The final examination consisted of 25 multiple-choice questions and required a 70% score. It was administered via Qualtrics, with two to three questions per session. As with most open online courses, we could not fully verify independent completion, and this is acknowledged as a limitation. Certificates were jointly issued by GCCHE and ASPHER and accredited by the Agency for Public Health Education Accreditation (APHEA) [18].

### Data analysis

All analyses were conducted on anonymized datasets. Descriptive statistics summarized participant characteristics, geographic distribution, attendance, certification status, and survey completion. On a 10-point Likert scale, percentage change was calculated as (post-course mean − pre-course mean)/pre-course mean × 100. Changes in self-reported confidence between pre- and post-course surveys were evaluated using paired Wilcoxon signed-rank tests.

Subgroup analyses were conducted by region (EU-27, EEA-32, and WHO-ER-53). These groupings reflect overlapping governance and policy contexts rather than mutually exclusive comparison groups. The analyses were descriptive and intended to assess consistency of results across policy-relevant European definitions. Regional groupings followed WHO classifications to ensure consistency with GCCHE course structure.

Analyses included 1029 matched respondents who attended at least seven sessions and completed both surveys. Within-person pre–post changes were tested using paired Wilcoxon signed-rank tests for each domain overall and within each regional grouping. We did not perform formal between-group hypothesis tests because regional groups overlap; subgroup results are presented descriptively. Statistical significance was defined as *P* < .05. All analyses were performed using R (version 4.3.2) and Microsoft Excel. Data visualizations were produced using the ggplot2 and plotly packages.

## Results

### Demographics and professional background

The 2025 European Climate Resilient Health Systems Course received 4432 registrations from 164 countries (27 from EU-27, 28 from EEA-32, and 42 from WHO ER-53—details in Table S1). Of these, 3018 participants from 153 countries attended at least one session, and 435 participated in all sessions. A total of 1456 participants from 133 countries (25 from EU-27, 26 from EEA-32, and 36 from WHO EURO) attended seven or more sessions, but 423 did not complete the final exam or post-course survey, and four did not pass the final exam. Ultimately, 1029 participants from 119 countries (24 countries from EU-27, 26 countries from EEA-32, and 33 countries from WHO EURO) attended at least 7 of the 10 sessions and completed the longitudinal survey; their data were used in this study’s impact analysis. Among these, 1025 participants from 119 countries fulfilled both the attendance and exam requirements and received a certificate. Respondent characteristics are shown in Table 1.

**Table 1:** Characteristics of matched survey respondents included in the longitudinal analysis, overall and by overlapping European regional grouping.^a^

	Survey respondent (*N* = 1029)		EU-27 (*n* = 345)		EEA-32 (*n* = 371)		WHO-ER-53 (*n* = 446)	
	#	%	#	%	#	%	#	%
Student status								
Student	635	61.7	174	50.4	190	51.2	250	56.1
Non-students	394	38.3	171	49.6	181	48.8	196	43.9
Previous formal education in climate and health								
No	520	50.5	185	53.6	196	52.8	231	51.8
Yes	509	49.5	160	46.4	175	47.2	215	48.2
Place of employment								
Government/Inter-governmental	278	27	55	15.9	58	15.6	87	19.5
Academic/Research Institution	375	36.4	178	51.6	192	51.8	213	47.8
Non-governmental organization/non-profit	177	17.2	26	7.5	31	8.4	43	9.6
Private sector	70	6.8	22	6.4	24	6.5	24	5.4
Other	129	12.5	64	18.6	66	17.8	79	17.7
Professional background								
Public Health	439	42.7	158	45.8	167	45	200	44.8
Medicine	155	15.1	71	20.6	77	20.8	88	19.7
Environmental Health	114	11.1	24	7	25	6.7	32	7.2
Public Health Policy	40	3.9	14	4.1	14	3.8	16	3.6
Nurse	32	3.1	9	2.6	9	2.4	13	2.9
Epidemiology	29	2.8	5	1.4	8	2.2	7	1.6
Natural/Physical Science	18	1.7	5	1.4	5	1.3	5	1.1
Consultant	16	1.6	7	2	8	2.2	9	2
Doctor	16	1.6	8	2.3	11	3	11	2.5
Health Administrators	11	1.1	3	0.9	4	1.1	5	1.1
Engineering/environmental Engineering	16	1.6	8	2.3	9	2.4	11	2.5
Others	143	13.9	42	12.2	34	9.2	49	11.0

a Data are presented as *n* (%). Percentages are column percentages. EU-27, EEA-32, and WHO-ER-53 are overlapping regional categories; participants from countries belonging to more than one grouping are counted in each relevant subgroup. Counts do not sum to the overall total. The overall column includes all matched respondents worldwide who attended at least seven sessions and completed both the pre- and post-course surveys.

Among the 1029 certificate participants, geographic representation was broad. The most represented countries were Italy (92; 8.9%), Ireland (72; 7.0%), Nigeria (72; 7.0%), United Kingdom (71; 6.9%), Kenya (67; 6.5%), Uganda (59; 5.7%), Ghana (43; 4.2%), France (29; 2.8%), Germany (26; 2.5%), and India (24; 2.3%). In total, 434 participants were from European countries, 345 from EU-27, 371 from EEA-32, and 446 from WHO ER-53 countries (Fig. 1). A full breakdown by country is provided in Appendix B.

**Figure 1:**
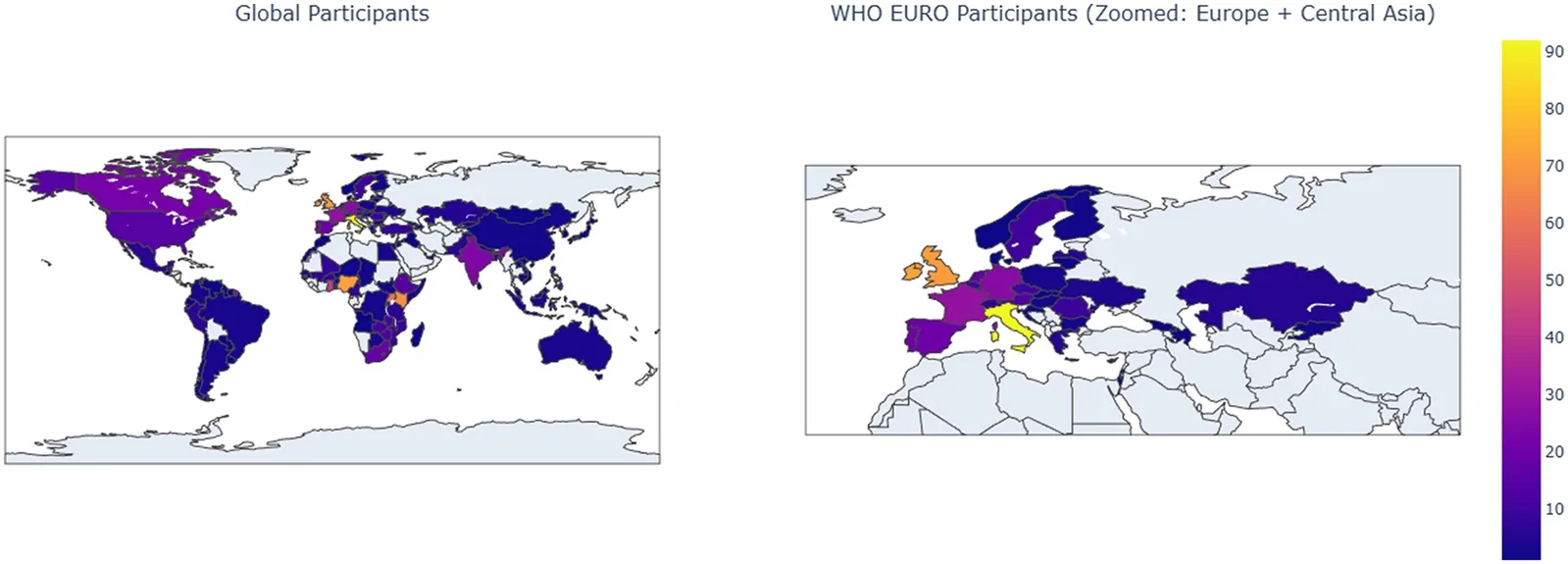
Global distribution of course participants (left, *n* = 1029) and participants from the 53 WHO European Region Member States (right, *n* = 446). The European panel is zoomed to highlight participant representation across the 53 countries of WHO EURO. Values correspond to the number of matched respondents included in the impact analysis per country.

Regarding professional status (Table 1), of the 1029 respondents to the demographic module, 394 (38.3%) self-identified as non-students, and 635 (61.7%) as students and 520 (50.5%) participants reported no previous formal education in climate and health, while 509 (49.5%) had some prior exposure. The results among regions did not vary (Table 1).

The largest proportion of participants were affiliated with academic and research institutions (375; 36.4%), followed by government or intergovernmental organizations (278; 27.0%) and non-governmental organizations (177; 17.2%). A small proportion was employed in the private sector (70; 6.8%) or other settings (129; 12.5%). Table 1 summarizes the top 11 professional backgrounds represented among participants, with rankings differing across regions. Overall, most of the participants’ self-declared disciplines clustered around Public Health (439; 42.7%) and Medicine (155; 15.1%), with notable representation from Environmental Health (114; 11.1%) and Public Health Policy (40; 3.9%).

### Pre- to post-course self-reported confidence gains

There was a marked improvement in participants’ confidence on a broad range of topics (Table 2 and Table S2). All paired pre–post changes were statistically significant (Wilcoxon signed-rank *P* < .001). Participants’ mean self-reported confidence in communicating about decarbonization and the resilience of health systems (Q1; Fig. 2) increased from 5.8 before the training to 8.5 after the training, representing a 46.2% improvement. Within the European division, these gains were sustained, with participants from EU-27 countries reporting an increase from 5.3 to 8.2 (53.7%), those from the EEA-32 rising from 5.3 to 8.2 (54.8%), and participants from the WHO-ER-53 improving from 5.4 to 8.2 (51.6%). Self-reported confidence in participating in vulnerability and adaptation assessments (Q2; Fig. 2 and Fig. S1) also increased substantially, from 5.5 to 8.1 (48.5%). Comparable trends were observed across Europe: EU-27 participants rose from 4.7 to 7.7 (61.8%), EEA-32 participants from 4.7 to 7.5 (59.7%), and those from the WHO ER-53 from 4.7 to 7.6 (60.3%).

**Figure 2:**
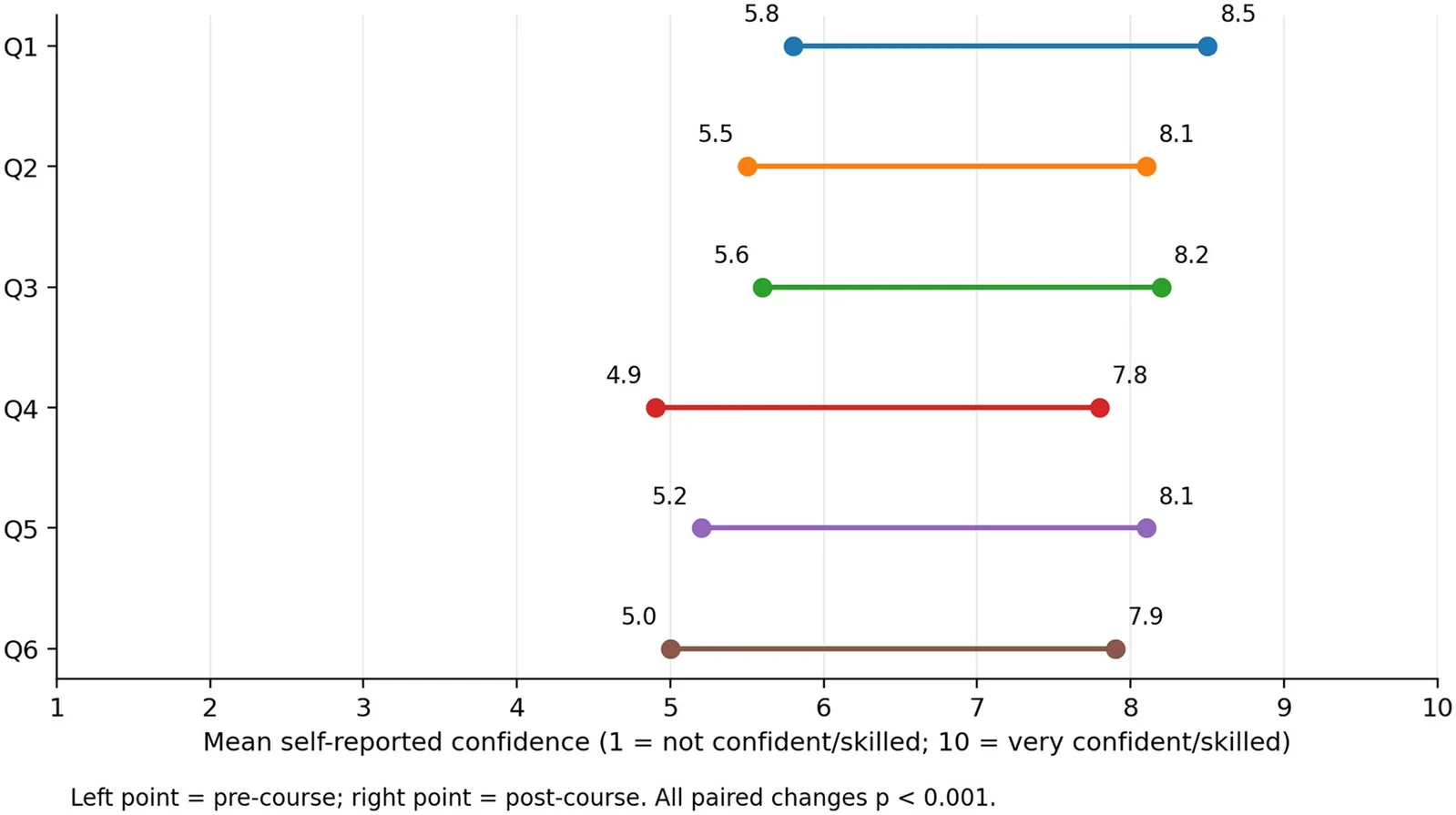
Pre- and post-course mean self-reported confidence across the six competency domains among matched respondents (*N* = 1029). Q1, communicating about health-system decarbonization and climate resilience; Q2, participating in vulnerability and adaptation assessments; Q3, contributing to resilience-building and reduction of health-system environmental footprints; Q4, applying vulnerability and adaptation frameworks; Q5, supporting community and health-system adaptation to climate-related threats; Q6, developing climate-resilience plans. All paired pre-post changes were statistically significant (*P* < .001).

**Table 2:** Pre- and post-course mean self-reported confidence across six climate-resilient health-system competency domains, overall and by overlapping European regional grouping.^a^

Competency	Overall pre -> post	Change	EU-27 pre -> post	EEA-32 pre -> post	WHO-ER-53 pre -> post	***P* value** *
Q1. Communicating about decarbonization and climate resilience	5.8 -> 8.5	+46.2%	5.3 -> 8.2	5.3 -> 8.2	5.4 -> 8.2	<.001
Q2. Participating in vulnerability and adaptation assessments	5.5 -> 8.1	+48.5%	4.7 -> 7.7	4.7 -> 7.5	4.7 -> 7.6	<.001
Q3. Contributing to resilience and reducing environmental footprints	5.6 -> 8.2	+45.6%	4.8 -> 7.1	5.1 -> 7.7	5.1 -> 7.7	<.001
Q4. Applying vulnerability and adaptation frameworks	4.9 -> 7.8	+58.5%	4.4 -> 7.2	4.3 -> 7.2	4.3 -> 7.3	<.001
Q5. Supporting community and health-system adaptation	5.2 -> 8.1	+55.9%	4.6 -> 7.6	4.6 -> 7.2	4.7 -> 7.6	<.001
Q6. Developing climate-resilience plans	5.0 -> 7.9	+59.3%	4.4 -> 7.5	4.3 -> 6.8	4.4 -> 7.3	<.001

a Values are means on a 10-point Likert scale. All analyses were done using a 10-point Likert scale. Percentage change is calculated as (post-course mean − pre-course mean)/pre-course mean × 100. EU-27, EEA-32, and WHO-ER-53 are overlapping regional groupings and should not be interpreted as independent comparison groups.

* Paired Wilcoxon signed-rank tests; the manuscript reports *P* < .001 for all six overall pre–post comparisons. Detailed answers to each question are provided in Table S2 (Appendix D).

Confidence in contributing to health system resilience and reducing environmental footprints (Q3; Fig. 2 and Fig. S2) increased from 5.6 to 8.2, corresponding to a 45.6% improvement. Among European subgroups, the mean self-reported confidence rose from 4.8 to 7.1 (59%) among EU-27 participants, from 5.1 to 7.7 (51.4%) among EEA-32 participants, and from 5.1 to 7.7 (52.8%) among those from the WHO-ER-53. Participants also reported substantial gains in applied skills. Mean scores for perceived ability to apply vulnerability and adaptation frameworks in health systems across different contexts (Q4; Fig. 2 and Fig. S3) improved from 4.9 to 7.8, a 58.5% increase. Within the European division, similar improvements were observed, with EU-27 participants increasing from 4.4 to 7.2 (66.3%), EEA-32 participants from 4.3 to 7.2 (67.6%), and WHO-ER-53 participants from 4.3 to 7.3 (68.3%).

Perceived confidence in supporting communities and healthcare systems to adapt to climate-related health threats (Q5; Fig. 2 and Fig. S4) increased from 5.2 to 8.1, reflecting a 55.9% improvement. Among European subgroups, EU-27 participants improved from 4.6 to 7.6 (63.7%), EEA-32 participants from 4.6 to 7.2 (57%), and WHO-ER-53 participants from 4.7 to 7.6 (63.4%). Finally, self-reported confidence in developing climate-resilient organizational plans (Q6; Fig. 2 and Fig. S5) demonstrated the largest relative gain, increasing from 5.0 to 7.9 (59.3%). Within the European division, EU-27 participants improved from 4.4 to 7.5 (73.9%), EEA-32 participants from 4.3 to 6.8 (58.2%), and WHO-ER-53 participants from 4.4 to 7.3 (67.2%).

## Discussion

This study evaluated the 2025 European Climate Resilient Health Systems Course delivered through the ASPHER–GCCHE partnership. It is among the largest systematic evaluations of an online climate and health training programme delivered in the WHO-ER-53 and provides results disaggregated by EU-27, EEA-32, and WHO-ER-53 groupings. The course engaged both students and professionals across sectors, generating evidence relevant to ministries of health, academic institutions, and multilateral agencies focused on workforce development. The open-access model also supported distributive equity by enabling participation from low- and middle-income countries. Finally, the curriculum’s combination of expert-led lectures and European case studies strengthened contextual relevance and applicability The course achieved significant pre–post improvements in Europe-based participants’ self-reported confidence across six competency domains, supporting the value of large-scale, competency-based online training for strengthening climate-resilient and sustainable health systems. The largest relative gains were observed for applied planning and adaptation domains, particularly developing climate-resilience plans and applying vulnerability and adaptation frameworks, while communication and health-system decarbonization domains also improved significantly. Although the course was designed around European frameworks, open access attracted substantial participation from LMICs. Because the curriculum used health-system concepts and case studies with broader relevance, these results suggest demand beyond Europe; however, the evaluation was not designed to assess contextual usefulness for LMIC learners specifically.

Despite growing attention to climate-resilient health systems, many national health systems remain insufficiently prepared for climate-related impacts [19–22]. Building climate-resilient health systems requires strengthening key system components, including a climate-ready health workforce [7].

The course achieved self-reported confidence gains, sustained across European subregions, suggesting that well-designed online education can strengthen climate-health competencies at scale. Compared with the 2024 European Climate and Health Responder Course, where mean relative gains ranged from 16.7% to 42.3% across eight longitudinal items, the present course showed somewhat larger gains across all six climate-resilient health system competency domains, ranging from 45.6% for contributing to resilience-building and reducing environmental footprints to 59.3% for developing climate-resilient organizational plans. Direct comparison with the Pan-American Climate Resilient Health Systems Course is limited because that evaluation used categorical outcomes and reported high baseline engagement with no statistically significant longitudinal changes [12, 13]. The high proportion of participants from academic and research institutions may indicate demand among institutions involved in training the future workforce, but our data cannot determine institutional readiness directly. The programme’s open-access format likely contributed to broad participation, particularly among health professionals from low- and middle-income countries, for whom cost is a documented barrier to continuing professional development. It is also possible that participants in Europe were better prepared to integrate the knowledge acquired in the course into previous training in other areas and hence gained more confidence than participants from other regions. Participation from African countries suggests that open-access European courses may meet a broader unmet demand for climate-health systems education. However, because case studies and policy framing were European, future adaptations should assess contextual relevance and learning needs in LMIC and African settings.

### Baseline capacity gaps and regional insights

Baseline self-reported confidence was low across most domains despite nearly half of participants reporting prior climate and health training, consistent with evidence that practical training in climate governance, health system resilience, and decarbonization remains patchy [4, 23, 24]. Many health professionals may be familiar with climate-related health impacts but lack structured preparation in systems-level adaptation planning and governance, including integration of mitigation–adaptation co-benefits [25]. Lower baseline scores relative to earlier European training focused on health impacts and emergency response may reflect the more technical, systems-oriented focus of this course [13].

We anticipated higher baseline self-reported confidence among participants from EU-27 countries given more developed governance frameworks, but self-reported confidence levels and gains were broadly similar across EU-27, EEA-32, and WHO-ER-53 subgroups. This suggests that workforce training gaps persist even in settings with strong policy infrastructures, indicating limited diffusion of climate-resilient health system education within undergraduate and postgraduate training.

The course also demonstrated substantial geographic reach. While earlier European initiatives were criticized for limited engagement beyond EU borders [26], this programme attracted registrants from 164 countries and strong participation from African countries. This may reflect GCCHE’s global network and the open-access model and highlights opportunities for transregional exchange and South–North learning.

### Implications for policy, education, and practice

The high enrolment and substantial pre–post gains suggest both strong demand for training and persistent needs for applied, systems-oriented education in climate-resilient and low-carbon health systems. Although health workers increasingly recognize climate change as a major determinant of health, structured training in climate-resilient and environmentally sustainable health systems remains limited [25, 27]. This limits implementation of the climate-resilient and low-carbon health system pillars promoted by WHO and embedded in emerging European and international policy frameworks.

The convergence in post-course self-reported confidence across European subregions suggests that scalable online programmes can act as equalizing mechanisms, reducing disparities in climate-health literacy. By building familiarity with frameworks for adaptation planning, resilience governance, sustainable financing, and health-sector decarbonization, MOOCs can help prepare the public health workforce to lead adaptation and sustainability transitions within health systems [28, 29]. Continued investment in competency-based training, alongside integration of climate and sustainability competencies into formal curricula, is likely essential to build capacity among public health professionals. Curricula for health professionals should move beyond general climate-health awareness and include: health-system vulnerability and adaptation assessments; climate-informed emergency preparedness and surveillance; low-carbon and environmentally sustainable care; governance, financing and accountability for resilient health systems; equity and climate justice; and communication skills for institutional change.

For policy and practice, WHO Europe and the European Environment Agency could consider this model as a scalable baseline training offer for climate-resilient and low-carbon health systems, while universities could embed similar content within formal curricula to address the persistent education gap identified in prior surveys [10]. Ministries of health may also benefit from co-branded and multilingual adaptations aligned with national adaptation planning needs.

Future evaluation should focus on whether confidence gains translate into sustained competencies and organizational change. Multilingual expansion, integration of micro-credentials within academic credit systems, and continued use of recognized accreditation approaches such as APHEA are priorities for next courses [17]. Finally, adding measures of ‘motivation to act’ and other indicators of transformative learning, used in related educational evaluations, could strengthen monitoring of workforce capacity building over time [30].

### Limitations

This evaluation relied on self-reported confidence, which is a useful proxy but does not directly measure real-world performance. Participation and survey completion were voluntary, raising the possibility of motivation and self-selection bias. Self-selection may have inflated observed confidence gains if highly motivated learners were more likely to complete both surveys, or if participants with supportive institutions had more time and infrastructure to attend sessions. Conversely, open enrolment may have included learners with limited prior training, lowering baseline confidence and increasing room for improvement. Attrition may also have resulted in overrepresentation of participants with stronger institutional support, more time availability, or more stable internet access. Participants’ factual participation and independent completion of the final exam could not be fully verified. The course’s English-only delivery may have limited participation from some non-English-speaking settings, including parts of Eastern Europe and Central Asia. These overlapping groups were selected to align with European governance and policy frameworks, not to infer causal differences. Future analyses could use mutually exclusive subregions or income categories to explore contextual variation.

## Conclusion

The 2025 European Climate Resilient Health Systems Course was associated with significant improvements in self-reported confidence across six competency domains among matched respondents. Its open-access, competency-based format, and broad participation suggest that online training can support workforce development for climate-resilient and low-carbon health systems. Future evaluations should assess whether confidence gains translate into sustained competencies, behaviour change and organizational practice.

## Supplementary Material

ckag193_Supplementary_Data

## Data Availability

The datasets generated and/or analysed during the current study are available from the corresponding author on reasonable request. Key points Competency-based online education can substantially strengthen climate-resilient health system skills among health professionals at scale. Large, open-access training programmes can reduce disparities in climate–health literacy across European regions and between high- and lower-resource settings. Baseline confidence in climate-resilient health system competencies remains low, even in countries with advanced climate and health policy frameworks. Integrating accredited climate and health training into formal education and professional development pathways could support implementation of climate-resilient and low-carbon health system policies.
