# Supplementary material for: Climate and health capacity building for climate-resilient health systems: a case study of the ASPHER-GCCHE European course
Source: Eur J Public Health. 2026 Oct 27;36(6):ckag193. doi: 10.1093/eurpub/ckag193 (PMC13713187; doi:10.1093/eurpub/ckag193)
Supplement: Supplementary file 1 [file ckag193_supplementary_data.zip › ejph-2026-02-om-0112-File004.docx]

**European Climate Resilient Health Systems Curriculum**

Start date: 28 January 2025

End date: 8 April 2025

Time: Tuesday, 12-1.30 CET/CEST

Frequency: weekly, exceptions: 04 March 2025 (Carnival)

Format: 90-min webinars = 50 min lecture + 2 case studies 15-min each

**Brief description:**

Health systems, including hospitals, are on the frontlines of climate change, bearing the costs of increasing disease burdens and more frequent extreme weather events while simultaneously inadvertently contributing to negative environmental and climate-related impacts (WHO, 2020). Climate change poses a myriad of threats to health systems and communities. These risks vary depending on geographic location, the intensity and magnitude of specific climate-related hazards (floods, droughts, severe storms, extreme heat, wildfires, disease outbreaks), the level of exposure to the hazard, and the level of existing vulnerability (e.g., access to mental healthcare). Health systems which have transformed their structural and operational practices to confront these challenges have seen enormous benefits in terms of increasing their resilience in the face of climate challenges while simultaneously reducing operational costs.

The goal of this 10-week course is to equip health professionals, federal, regional and local health systems planners, hospital administrators, emergency planners, sustainability officers, public health officials, and others with the knowledge and tools needed to prepare health systems for climate change. This includes how to apply best practices to assess health system vulnerabilities and adaptation options, measure and monitor the climate resilience and environmental sustainability of health systems, and undertake health system greening, all while integrating health equity and undertaking broad stakeholder engagement.

**Overall learning objectives of course:**

- Articulate/communicate to key partners and the public the unique challenges health systems (including primary and secondary healthcare) face in a changing climate
- Apply methodology and frameworks for assessing vulnerability and adaptation for health systems in any context
- Apply approaches for measuring and monitoring the environmental sustainability of health systems and health co-benefits of transitioning to green health systems
- Apply a health equity lens into efforts to address health system decarbonization and climate resilience
- Develop an initial plan to increase the resilience of the health system vis-à-vis the estimated climate impact on their population and to engage with key partners to achieve meaningful adaptation.

**Audience:**

The expected audience includes health systems planners, hospital administrators, health facility board members, long-term care/community care professionals, business continuity officers, emergency planners, sustainability officers, public health officials, health practitioners, and paramedics

**General Course Structure:**

The course will consist of **once-weekly** live-virtual sessions (90 minutes), offered in English. Each session will consist of 50 minutes of “theory/foundation” followed by 1-2 case studies, representing instances across Europe. There will be a live question and answer which will be monitored by the program team and questions will be consolidated and addressed to expert lecturers. Resources such as frameworks and suggested readings will be provided to all course participants.

**SESSION 1: Healthcare systems in a changing climate: Overview and introduction to course**

**Description:** This session will introduce participants to the reciprocal relationship between climate change and health systems. Health systems contribute to climate change and environmental degradation. Climate change poses substantial risks to health systems both acutely during extreme weather events and chronically by increasing need for healthcare due to climate-sensitive diseases.

**Learning outcomes:**

1. Articulate acute and slow-onset climate change risks to health and healthcare systems and the role of health system capacity and resilience in buffering impacts
2. Describe the contribution of health care systems to global warming and associated negative health externalities in Europe and globally
3. Describe fundamental health system adaptation processes and steps to prepare for climate change
4. Identify the roles of the following key stakeholders in the health adaptation process (health system planners, clinicians, public health officials, civil society partners, researchers, decision makers outside health sector)

**SESSION 2: Methods for assessing vulnerability and adaptation of health systems**

**Description:** This session explores climate change and health vulnerability assessments as critical tools for building health system resilience. Using European case studies, participants will learn to identify at-risk populations, evaluate current and future health risks, and establish baselines for monitoring changes. The session also highlights strategies for adapting policies and fostering cross-sector collaborations to mitigate risks, alongside practical guidance on using frameworks and methods for effective assessments.

**Learning outcomes:**

1. Describe the concept of a climate change and health vulnerability and adaptation assessment and how such assessments form the basis of health system preparedness and resilience, including:
   a. Improving evidence and understanding of links between weather/climate and health outcomes.
   b. Identify populations most at risk from climate change impacts.
   c. Provide robust evidence of current and future projected health risks
   d. Establish a baseline to use to monitor changes in future risks and associated policies and programs.
   e. Support the development of needed collaborations with decision makers in other sectors (e.g., water, infrastructure, transportation, agriculture, urban planning, energy) to reduce risks to health.
   f. Identifying adaptation options for adjusting existing policies and programs and for developing new ones to prevent and reduce the severity of future risks.
2. Identify and utilise available frameworks and methods to undertake a climate change and health vulnerability and adaptation assessment

**SESSION 3: Health system emergency prevention, preparedness and response**

**Description:** This session focuses on strengthening health system resilience to climate-related hazards through vulnerability assessments, adaptation strategies, and innovative tools like climate stress testing. Participants will explore methods to evaluate health system vulnerabilities, prepare for future climate impacts, and implement resilience-building measures. European case studies will highlight successful approaches to enhancing the readiness of healthcare facilities for extreme weather and other climate challenges.

**Learning outcomes:**

1. Describe how to assess the vulnerability of health systems and facilities to climate change impacts and identify adaptation options using new tools and methods
2. Explain the concept of “climate stress testing for health systems and facilities” as it relates to preparing for future climate change impacts
3. Provide examples of the ways in which healthcare facilities can become more resilient in the face of increasingly severe and/or frequent climate-related hazards, such as extreme weather events

**SESSION 4: Health and economic benefits of sustainable health systems**

**Description:** This module explores the intersection of health systems strengthening and climate action, highlighting co-benefits for mitigation and ecosystem preservation. Learners will examine frameworks for integrating climate resilience into health system performance assessments. Through case studies, the module illustrates strengthening primary health care as a key health policy will reduce environmental impact, lower healthcare costs, and enhance population health.

**Learning outcomes:**

1. Explain how strengthening primary health care and health systems can yield co-benefits for climate mitigation and ecosystem preservation.
2. Discuss approaches for embedding climate resilience into health system performance assessments.
3. Provide examples of policies and strategies within the health sector that reduce environmental impact, lower healthcare costs, and improve population health.

**SESSION 5: Opportunities and guidance for lowering the carbon footprint of health systems**

**Description:** This session examines the greenhouse gas (GHG) emissions generated by health systems, categorised into scopes 1–3, and explores their sources and impacts. Participants will learn the basics of life-cycle analysis, review current estimates of health sector emissions, and identify opportunities to decarbonize and reduce emissions across all scopes. Case studies from Europe will illustrate practical strategies for creating more sustainable and climate-resilient healthcare systems.

**Learning outcomes:**

1. Define scope 1-3 greenhouse gas emissions from health systems and analyze the contributions to each
2. Describe the theoretical basis of a life-cycle analysis
3. Examine current estimates of GHG emissions from health systems
4. Identify opportunities to reduce emissions within each scope and associated examples

**SESSION 6: Health early warning systems and surveillance in a changing climate**

**Description:** This session explores the role of early warning systems in mitigating climate-related health risks, focusing on their core components—data collection, risk assessment, and communication strategies. Participants will learn to identify climate-sensitive health indicators and integrate them into surveillance systems for timely public health responses. Using European case studies, the session highlights ways to enhance surveillance frameworks with climate projections, stakeholder engagement, and technological innovations to strengthen community resilience and preparedness.

**Learning outcomes:**

1. Explain the core components of early warning systems, including data collection, risk assessment, and communication strategies, and how these systems mitigate health risks in the context of climate change.
2. Identify and evaluate climate-sensitive health indicators (e.g., vector-borne diseases, heat-related illnesses) and their integration into surveillance systems for timely and effective public health responses.
3. Explain how to enhance health surveillance frameworks that incorporate climate change projections, stakeholder engagement, and technological innovations to improve community resilience and preparedness.

**SESSION 7: Incorporating sustainability into Health Technology Assessment**

**Description:** This session will describe different methods and frameworks currently under development to allow integrating environmental considerations into Health Technology Assessments as well as their strengths and limitations. Participants will also learn about ethical dilemmas related to balancing individual versus collective/societal health and wellbeing benefits.

**Learning outcomes:**

1. Analyse the characteristics, merits and limitations of methods and frameworks currently under development to allow integrating environmental considerations into Health Technology Assessments.
2. Discuss the ethical dilemmas associated with taking into account environmental considerations in decisions about health technologies.
3. Explain how financial tools can be used to encourage progress towards sustainable and resilient health systems.

**SESSION 8: Measuring, monitoring and evaluating health systems resilience**

**Description:** This session examines the key concepts and frameworks for assessing climate-resilient, low-carbon, and carbon-neutral health systems. Participants will explore indicators for measuring resilience and delve into priority areas for monitoring and evaluation. European case studies will illustrate practical applications, highlighting strategies for strengthening health systems in the face of climate change challenges.

**Learning outcomes:**

1. Define a “climate resilient health system”
2. Define a low carbon and carbon neutral health system
3. Describe the current indicators for measuring the climate resilience of health systems
4. Describe the areas of focus for monitoring and evaluation of climate-resilient health facilities
   a. Financing
   b. Health workforce
   c. Service delivery
   d. Essential medical products and technologies
   e. Health information systems
   f. Leadership and governance
   g. Health Emergency Planning

**SESSION 9: Climate justice and governance**

**Description:** This session will explore how climate change and planetary health should guide the pursuit of universal health coverage, which is one of the UN sustainable development goals. It will also consider the importance of multilateral funding to enable achieving this ambitious goal from the lens of climate justice. Finally, participants will learn about the complex governance mechanisms involving national and international entities, including the role of the WHO and philanthropic organizations.

**Learning outcomes:**

1. Discuss how climate change and sustainability impact on achieving universal health coverage and the opportunity to develop sustainable health systems in LMIC.
2. Discuss the role of finance mechanisms, particularly multilateral funding, in achieving universal health coverage from the perspective of climate justice.
3. Analyze the complex governance mechanisms involving multiple stakeholders globally.

**SESSION 10: Health communication and education in a changing climate**

**Description:** This session explores the crucial role of health communication and education in driving behavioural changes related to climate adaptation and GHG mitigation. Participants will learn strategies for effectively communicating climate health risks to diverse stakeholders, emphasising the importance of audience segmentation and message tailoring. The session also examines the potential mental health impacts of climate communications and offers solutions to overcome local and national challenges in health system adaptation, with case studies from Europe showcasing successful engagement approaches.

**Learning outcomes:**

1. Describe the importance of climate change communication and education about health risks in motivating needed behavioural changes related to adaptation and GHG mitigations
2. Identify key strategies to communicate climate change health risks to stakeholders and the public
3. Explain the importance of audience identification, segmentation and message tailoring when communicating climate change
4. Identify the potentially damaging or empowering effects of climate change communications on mental health
5. Identify local and national challenges to undertaking health system adaptation and assess possible communications and engagement solutions
