# Supplementary material for: Climate and health capacity building for climate-resilient health systems: a case study of the ASPHER-GCCHE European course
Source: Eur J Public Health. 2026 Oct 27;36(6):ckag193. doi: 10.1093/eurpub/ckag193 (PMC13713187; doi:10.1093/eurpub/ckag193)
Supplement: Supplementary file 1 [file ckag193_supplementary_data.zip › ejph-2026-02-om-0112-File010.docx]

**Table S1. Geographic Distribution of Participants**

| Number | Country | nParticipant | Percentage |
| --- | --- | --- | --- |
| 1 | Italy | 92 | 8.9 |
| 2 | Ireland | 72 | 7 |
| 3 | Nigeria | 72 | 7 |
| 4 | United-Kingdom | 71 | 6.9 |
| 5 | Kenya | 67 | 6.5 |
| 6 | Uganda | 59 | 5.7 |
| 7 | Ghana | 43 | 4.2 |
| 8 | France | 29 | 2.8 |
| 9 | Germany | 26 | 2.5 |
| 10 | India | 24 | 2.3 |
| 11 | Canada | 22 | 2.1 |
| 12 | Portugal | 20 | 1.9 |
| 13 | Spain | 20 | 1.9 |
| 14 | Netherlands | 17 | 1.7 |
| 15 | South-Africa | 18 | 1.7 |
| 16 | UnitedStates | 15 | 1.5 |
| 17 | Ethiopia | 14 | 1.4 |
| 18 | Austria | 13 | 1.3 |
| 19 | Philippines | 13 | 1.3 |
| 20 | Zimbabwe | 13 | 1.3 |
| 21 | Belgium | 11 | 1.1 |
| 22 | Zambia | 11 | 1.1 |
| 23 | Czechia | 10 | 1 |
| 24 | Sweden | 10 | 1 |
| 25 | Lesotho | 9 | 0.9 |
| 26 | Mozambique | 9 | 0.9 |
| 27 | Romania | 9 | 0.9 |
| 28 | Malawi | 8 | 0.8 |
| 29 | Mali | 8 | 0.8 |
| 30 | Turkey | 8 | 0.8 |
| 31 | Burkina-Faso | 7 | 0.7 |
| 32 | Cameroon | 7 | 0.7 |
| 33 | Peru | 7 | 0.7 |
| 34 | Switzerland | 7 | 0.7 |
| 35 | Trinidad-Tobago | 7 | 0.7 |
| 36 | Bangladesh | 6 | 0.6 |
| 37 | Latvia | 6 | 0.6 |
| 38 | Mexico | 6 | 0.6 |
| 39 | Pakistan | 6 | 0.6 |
| 40 | Tanzania | 6 | 0.6 |
| 41 | Bahamas | 5 | 0.5 |
| 42 | Greece | 5 | 0.5 |
| 43 | Kazakhstan | 5 | 0.5 |
| 44 | Singapore | 5 | 0.5 |
| 45 | Botswana | 4 | 0.4 |
| 46 | Indonesia | 4 | 0.4 |
| 47 | Moldova | 4 | 0.4 |
| 48 | Senegal | 4 | 0.4 |
| 49 | Somalia | 4 | 0.4 |
| 50 | Ukraine | 4 | 0.4 |
| 51 | Argentina | 3 | 0.3 |
| 52 | Australia | 3 | 0.3 |
| 53 | Brazil | 3 | 0.3 |
| 54 | Chile | 3 | 0.3 |
| 55 | Colombia | 3 | 0.3 |
| 56 | Congo | 3 | 0.3 |
| 57 | Egypt | 3 | 0.3 |
| 58 | Gambia | 3 | 0.3 |
| 59 | Poland | 3 | 0.3 |
| 60 | St.Kitts | 3 | 0.3 |
| 61 | Belize | 2 | 0.2 |
| 62 | Brunei | 2 | 0.2 |
| 63 | Bulgaria | 2 | 0.2 |
| 64 | Cambodia | 2 | 0.2 |
| 65 | Chad | 2 | 0.2 |
| 66 | China | 2 | 0.2 |
| 67 | Djibouti | 2 | 0.2 |
| 68 | Finland | 2 | 0.2 |
| 69 | Guyana | 2 | 0.2 |
| 70 | Hungary | 2 | 0.2 |
| 71 | Jamaica | 2 | 0.2 |
| 72 | Kyrgyzstan | 2 | 0.2 |
| 73 | Mauritius | 2 | 0.2 |
| 74 | Niger | 2 | 0.2 |
| 75 | Rwanda | 2 | 0.2 |
| 76 | South-Sudan | 2 | 0.2 |
| 77 | United-Arab_Emirates | 2 | 0.2 |
| 79 | Angola | 1 | 0.1 |
| 80 | Azerbaijan | 1 | 0.1 |
| 81 | Barbados | 1 | 0.1 |
| 82 | Benin | 1 | 0.1 |
| 83 | Croatia | 1 | 0.1 |
| 84 | Cyprus | 1 | 0.1 |
| 85 | Denmark | 1 | 0.1 |
| 86 | Ecuador | 1 | 0.1 |
| 87 | Eswatini | 1 | 0.1 |
| 88 | Georgia | 1 | 0.1 |
| 89 | Grenada | 1 | 0.1 |
| 90 | Guatemala | 1 | 0.1 |
| 91 | Guinea-Bissau | 1 | 0.1 |
| 92 | Haiti | 1 | 0.1 |
| 93 | Iraq | 1 | 0.1 |
| 94 | Israel | 1 | 0.1 |
| 95 | Japan | 1 | 0.1 |
| 96 | Jordan | 1 | 0.1 |
| 97 | Laos | 1 | 0.1 |
| 98 | Lithuania | 1 | 0.1 |
| 99 | Luxembourg | 1 | 0.1 |
| 100 | Madagascar | 1 | 0.1 |
| 101 | Malaysia | 1 | 0.1 |
| 102 | Mongolia | 1 | 0.1 |
| 103 | Morocco | 1 | 0.1 |
| 104 | Nepal | 1 | 0.1 |
| 105 | Norway | 1 | 0.1 |
| 106 | Panama | 1 | 0.1 |
| 107 | Papua-New-Guinea | 1 | 0.1 |
| 108 | Paraguay | 1 | 0.1 |
| 109 | Seychelles | 1 | 0.1 |
| 110 | Slovakia | 1 | 0.1 |
| 111 | South-Korea | 1 | 0.1 |
| 112 | SriLanka | 1 | 0.1 |
| 113 | Suriname | 1 | 0.1 |
| 114 | São-Tome | 1 | 0.1 |
| 115 | Taiwan | 1 | 0.1 |
| 116 | Togo | 1 | 0.1 |
| 117 | Uruguay | 1 | 0.1 |
| 118 | Venezuela | 1 | 0.1 |
| 119 | Vietnam | 1 | 0.1 |
