# Supplementary material for: Climate and health capacity building for climate-resilient health systems: a case study of the ASPHER-GCCHE European course
Source: Eur J Public Health. 2026 Oct 27;36(6):ckag193. doi: 10.1093/eurpub/ckag193 (PMC13713187; doi:10.1093/eurpub/ckag193)
Supplement: Supplementary file 1 [file ckag193_supplementary_data.zip › ejph-2026-02-om-0112-File011.docx]

**Table S2.** Distribution of responses to longitudinal survey questions among participants who attended seven or more sessions, overall and stratified by geographic group (EU-27 countries, EEA-32 countries, and the WHO European Region). Responses were scored on a 10-point Likert scale ranging from 1 (“Not confident” or “Not skilled”) to 10 (“Very confident” or “Very skilled”). *Pre-course and post-course responses were compared for each question using the Wilcoxon signed-rank test. All questions showed statistically significant improvements from pre- to post-course, with p < 0.001 for every comparison.*

| **All participants (N=1029)** | | |  | **EU-27 (n=345)** | |  | **EEA-32 (n=332)** | |  | **WHO EURO (n=446)** | |
| --- | --- | --- | --- | --- | --- | --- | --- | --- | --- | --- | --- |
| **Rating** | **PRE_Number of response (%)** | **POST- Number of responses (%)** |  | **PRE_Number of response (%)** | **POST- Number of responses (%)** |  | **PRE_Number of response (%)** | **POST- Number of responses (%)** |  | **PRE_Number of response (%)** | **POST- Number of responses (%)** |
| **Q1 - “To what extent do you feel confident communicating with work colleagues or members of your community about the need to reduce the carbon footprint and strengthen the resilience of health systems?** | | | | | | | | | | | |
| **1 - Not confident** | 40 (3.9%) | 0 (0.0%) |  | 20 (5.8%) | 0 (0.0%) |  | 23 (6.2%) | 0 (0.0%) |  | 22 (4.9%) | 0 (0.0%) |
| **2** | 42 (4.1%) | 0 (0.0%) |  | 21 (6.1%) | 0 (0.0%) |  | 23 (6.2%) | 0 (0.0%) |  | 23 (5.2%) | 0 (0.0%) |
| **3** | 94 (9.1%) | 4 (0.4%) |  | 41 (11.9%) | 2 (0.6%) |  | 45 (12.1%) | 2 (0.5%) |  | 58 (13.0%) | 2 (0.4%) |
| **4** | 144 (14.0%) | 3 (0.3%) |  | 57 (16.5%) | 2 (0.6%) |  | 62 (16.7%) | 3 (0.8%) |  | 74 (16.6%) | 2 (0.4%) |
| **5** | 169 (16.4%) | 15 (1.5%) |  | 46 (13.3%) | 7 (2.0%) |  | 51 (13.7%) | 9 (2.4%) |  | 61 (13.7%) | 8 (1.8%) |
| **6** | 143 (13.9%) | 57 (5.5%) |  | 48 (13.9%) | 26 (7.5%) |  | 49 (13.2%) | 30 (8.1%) |  | 62 (13.9%) | 36 (8.1%) |
| **7** | 145 (14.1%) | 127 (12.3%) |  | 41 (11.9%) | 63 (18.3%) |  | 44 (11.9%) | 64 (17.3%) |  | 52 (11.7%) | 73 (16.4%) |
| **8** | 95 (9.2%) | 279 (27.1%) |  | 41 (11.9%) | 102 (29.6%) |  | 42 (11.3%) | 110 (29.6%) |  | 52 (11.7%) | 139 (31.2%) |
| **9** | 41 (4.0%) | 248 (24.1%) |  | 10 (2.9%) | 70 (20.3%) |  | 11 (3.0%) | 76 (20.5%) |  | 13 (2.9%) | 93 (20.9%) |
| **10- Very confident** | 116 (11.3%) | 296 (28.8%) |  | 20 (5.8%) | 73 (21.2%) |  | 21 (5.7%) | 77 (20.8%) |  | 29 (6.5%) | 93 (20.9%) |
|  |  |  |  |  |  |  |  |  |  |  |  |
| **Q2 - To what extent do you feel confident in your ability to participate in health system vulnerability and adaptation assessments?** | | | | | | | | | | | |
| **1 - Not confident** | 54 (5.2%) | 0 (0.0%) |  | 26 (7.5%) | 0 (0.0%) |  | 29 (7.8%) | 0 (0.0%) |  | 35 (7.8%) | 0 (0.0%) |
| **2** | 74 (7.2%) | 3 (0.3%) |  | 38 (11.0%) | 2 (0.6%) |  | 42 (11.3%) | 3 (0.8%) |  | 48 (10.8%) | 2 (0.4%) |
| **3** | 103 (10.0%) | 4 (0.4%) |  | 44 (12.8%) | 3 (0.9%) |  | 48 (12.9%) | 3 (0.8%) |  | 59 (13.2%) | 4 (0.9%) |
| **4** | 140 (13.6%) | 10 (1.0%) |  | 58 (16.8%) | 6 (1.7%) |  | 65 (17.5%) | 7 (1.9%) |  | 74 (16.6%) | 8 (1.8%) |
| **5** | 166 (16.1%) | 36 (3.5%) |  | 52 (15.1%) | 16 (4.6%) |  | 55 (14.8%) | 20 (5.4%) |  | 69 (15.5%) | 22 (4.9%) |
| **6** | 156 (15.2%) | 100 (9.7%) |  | 54 (15.7%) | 54 (15.7%) |  | 55 (14.8%) | 59 (15.9%) |  | 66 (14.8%) | 66 (14.8%) |
| **7** | 140 (13.6%) | 174 (16.9%) |  | 36 (10.4%) | 76 (22.0%) |  | 38 (10.2%) | 81 (21.8%) |  | 48 (10.8%) | 104 (23.3%) |
| **8** | 70 (6.8%) | 262 (25.5%) |  | 15 (4.3%) | 97 (28.1%) |  | 15 (4.0%) | 103 (27.8%) |  | 19 (4.3%) | 124 (27.8%) |
| **9** | 24 (2.3%) | 215 (20.9%) |  | 6 (1.7%) | 52 (15.1%) |  | 7 (1.9%) | 54 (14.6%) |  | 9 (2.0%) | 65 (14.6%) |
| **10- Very confident** | 102 (9.9%) | 225 (21.9% |  | 16 (4.6%) | 39 (11.3%) |  | 17 (4.6%) | 41 (11.1%) |  | 19 (4.3%) | 51 (11.4%) |
|  |  |  |  |  |  |  |  |  |  |  |  |
| **Q3 - To what extent do you feel confident in your ability to participate in reducing the environmental footprint of and increasing the resilience of your health system?** | | | | | | | | | | | |
| **1 - Not confident** | 40 (3.9%) | 0 (0.0%) |  | 23 (6.7%) | 0 (0.0%) |  | 26 (7.0%) | 0 (0.0%) |  | 27 (6.1%) | 0 (0.0%) |
| **2** | 46 (4.5%) | 2 (0.2%) |  | 31 (9.0%) | 1 (0.3%) |  | 31 (8.4%) | 1 (0.3%) |  | 35 (7.8%) | 2 (0.4%) |
| **3** | 114 (11.1%) | 4 (0.4%) |  | 43 (12.5%) | 3 (0.9%) |  | 50 (13.5%) | 3 (0.8%) |  | 59 (13.2%) | 3 (0.7%) |
| **4** | 140 (13.6%) | 12 (1.2%) |  | 49 (14.2%) | 9 (2.6%) |  | 56 (15.1%) | 10 (2.7%) |  | 63 (14.1%) | 10 (2.2%) |
| **5** | 177 (17.2%) | 29 (2.8%) |  | 55 (15.9%) | 12 (3.5%) |  | 59 (15.9%) | 15 (4.0%) |  | 75 (16.8%) | 15 (3.4%) |
| **6** | 151 (14.7%) | 91 (8.8%) |  | 52 (15.1%) | 46 (13.3%) |  | 54 (14.6%) | 51 (13.7%) |  | 63 (14.1%) | 63 (14.1%) |
| **7** | 155 (15.1%) | 173 (16.8%) |  | 46 (13.3%) | 73 (21.2%) |  | 48 (12.9%) | 79 (21.3%) |  | 61 (13.7%) | 94 (21.1%) |
| **8** | 74 (7.2%) | 248 (24.1%) |  | 24 (7.0%) | 88 (25.5%) |  | 25 (6.7%) | 94 (25.3%) |  | 34 (7.6%) | 115 (25.8%) |
| **9** | 37 (3.6%) | 227 (22.1%) |  | 9 (2.6%) | 57 (16.5%) |  | 9 (2.4%) | 61 (16.4%) |  | 13 (2.9%) | 78 (17.5%) |
| **10- Very confident** | 95 (9.2%) | 243 (23.6%) |  | 13 (3.8%) | 56 (16.2%) |  | 13 (3.5%) | 57 (15.4%) |  | 16 (3.6%) | 66 (14.8%) |
|  |  |  |  |  |  |  |  |  |  |  |  |
| **Q4 - To what extent do you feel skilled in applying vulnerability and adaptation frameworks for health systems in diverse contexts?** | | | | | | | | | | | |
| **1 - Not skilled** | 77 (7.5%) | 1 (0.1%) |  | 38 (11.0%) | 0 (0.0%) |  | 41 (11.1%) | 0 (0.0%) |  | 27 (6.1%) | 0 (0.0%) |
| **2** | 96 (9.3%) | 2 (0.2%) |  | 48 (13.9%) | 2 (0.6%) |  | 51 (13.7%) | 2 (0.5%) |  | 35 (7.8%) | 2 (0.4%) |
| **3** | 132 (12.8%) | 9 (0.9%) |  | 48 (13.9%) | 6 (1.7%) |  | 56 (15.1%) | 6 (1.6%) |  | 59 (13.2%) | 3 (0.7%) |
| **4** | 145 (14.1%) | 20 (1.9%) |  | 52 (15.1%) | 14 (4.1%) |  | 57 (15.4%) | 15 (4.0%) |  | 63 (14.1%) | 10 (2.2%) |
| **5** | 176 (17.1%) | 46 (4.5%) |  | 53 (15.4%) | 30 (8.7%) |  | 57 (15.4%) | 34 (9.2%) |  | 75 (16.8%) | 15 (3.4%) |
| **6** | 140 (13.6%) | 131 (12.7%) |  | 47 (13.6%) | 63 (18.3%) |  | 47 (12.7%) | 70 (18.9%) |  | 63 (14.1%) | 63 (14.1%) |
| **7** | 124 (12.1%) | 190 (18.5%) |  | 30 (8.7%) | 67 (19.4%) |  | 32 (8.6%) | 73 (19.7%) |  | 61 (13.7%) | 94 (21.1%) |
| **8** | 55 (5.3%) | 259 (25.2%) |  | 13 (3.8%) | 85 (24.6%) |  | 14 (3.8%) | 88 (23.7%) |  | 34 (7.6%) | 115 (25.8%) |
| **9** | 32 (3.1%) | 189 (18.4%) |  | 7 (2.0%) | 40 (11.6%) |  | 7 (1.9%) | 44 (11.9%) |  | 13 (2.9%) | 78 (17.5%) |
| **10 - Very skilled** | 52 (5.1%) | 182 (17.7%) |  | 9 (2.6%) | 38 (11.0%) |  | 9 (2.4%) | 39 (10.5%) |  | 16 (3.6%) | 66 (14.8%) |
|  |  |  |  |  |  |  |  |  |  |  |  |
| **Q5 - To what extent do you feel skilled in helping your community and healthcare system adapt to the health threats posed by climate change?** | | | | | | | | | | | |
| **1 - Not skilled** | 53 (5.2%) | 0 (0.0%) |  | 25 (7.2%) | 0 (0.0%) |  | 30 (8.1%) | 0 (0.0%) |  | 32 (7.2%) | 0 (0.0%) |
| **2** | 74 (7.2%) | 1 (0.1%) |  | 42 (12.2%) | 0 (0.0%) |  | 44 (11.9%) | 0 (0.0%) |  | 49 (11.0%) | 0 (0.0%) |
| **3** | 124 (12.1%) | 5 (0.5%) |  | 55 (15.9%) | 5 (1.4%) |  | 61 (16.4%) | 5 (1.3%) |  | 71 (15.9%) | 5 (1.1%) |
| **4** | 152 (14.8%) | 12 (1.2%) |  | 51 (14.8%) | 7 (2.0%) |  | 57 (15.4%) | 8 (2.2%) |  | 67 (15.0%) | 9 (2.0%) |
| **5** | 191 (18.6%) | 26 (2.5%) |  | 50 (14.5%) | 13 (3.8%) |  | 54 (14.6%) | 16 (4.3%) |  | 69 (15.5%) | 15 (3.4%) |
| **6** | 138 (13.4%) | 86 (8.4%) |  | 51 (14.8%) | 48 (13.9%) |  | 52 (14.0%) | 51 (13.7%) |  | 63 (14.1%) | 59 (13.2%) |
| **7** | 134 (13.0%) | 186 (18.1%) |  | 33 (9.6%) | 89 (25.8%) |  | 34 (9.2%) | 97 (26.1%) |  | 47 (10.5%) | 110 (24.7%) |
| **8** | 59 (5.7%) | 277 (26.9%) |  | 19 (5.5%) | 93 (27.0%) |  | 20 (5.4%) | 98 (26.4%) |  | 24 (5.4%) | 130 (29.1%) |
| **9** | 41 (4.0%) | 214 (20.8%) |  | 8 (2.3%) | 46 (13.3%) |  | 8 (2.2%) | 50 (13.5%) |  | 12 (2.7%) | 66 (14.8%) |
| **10 - Very skilled** | 63 (6.1%) | 222 (21.6%) |  | 11 (3.2%) | 44 (12.8%) |  | 11 (3.0%) | 46 (12.4%) |  | 12 (2.7%) | 52 (11.7%) |
|  |  |  |  |  |  |  |  |  |  |  |  |
| **Q6 - To what extent do you feel skilled in developing climate resilience plans for organizations?** | | | | | | | | | | | |
| **1 - Not skilled** | 89 (8.6%) | 0 |  | 40 (11.6%) | 0 (0.0%) |  | 45 (12.1%) | 0 (0.0%) |  | 51 (11.4%) | 0 (0.0%) |
| **2** | 89 (8.6%) | 8 (0.8%) |  | 48 (13.9%) | 6 (1.7%) |  | 50 (13.5%) | 7 (1.9%) |  | 54 (12.1%) | 6 (1.3%) |
| **3** | 127 (12.3%) | 7 (0.7%) |  | 48 (13.9%) | 5 (1.4%) |  | 56 (15.1%) | 6 (1.6%) |  | 66 (14.8%) | 5 (1.1%) |
| **4** | 138 (13.4%) | 12 (1.2%) |  | 52 (15.1%) | 10 (2.9%) |  | 56 (15.1%) | 10 (2.7%) |  | 66 (14.8%) | 11 (2.5%) |
| **5** | 169 (16.4%) | 45 (4.4%) |  | 52 (15.1%) | 27 (7.8%) |  | 54 (14.6%) | 29 (7.8%) |  | 67 (15.0%) | 31 (7.0%) |
| **6** | 147 (14.3%) | 123 (12%) |  | 47 (13.6%) | 64 (18.6%) |  | 50 (13.5%) | 69 (18.6%) |  | 62 (13.9%) | 78 (17.5%) |
| **7** | 127 (12.3%) | 183 (17.8%) |  | 27 (7.8%) | 68 (19.7%) |  | 28 (7.5%) | 73 (19.7%) |  | 37 (8.3%) | 100 (22.4%) |
| **8** | 55 (5.3%) | 261 (25.4%) |  | 15 (4.3%) | 82 (23.8%) |  | 16 (4.3%) | 86 (23.2%) |  | 24 (5.4%) | 106 (23.8%) |
| **9** | 32 (3.1%) | 186 (18.1%) |  | 8 (2.3%) | 46 (13.3%) |  | 8 (2.2%) | 50 (13.5%) |  | 11 (2.5%) | 58 (13.0%) |
| **10 - Very skilled** | 56 (5.4%) | 204 (19.8%) |  | 8 (2.3%) | 37 (10.7%) |  | 8 (2.2%) | 41 (11.1%) |  | 8 (1.8%) | 51 (11.4%) |
